# Evidence of microRNAs origination from chloroplast genome and their role in regulating Photosystem II protein N (psbN) mRNA

**DOI:** 10.5114/bta.2024.135639

**Published:** 2024-03-29

**Authors:** Asha Anand, Shailja Chauhan, Aparna Chodon, Kavitha Velayudha Vimala Kumar, S. Saravanakumar, Gopal Pandi

**Affiliations:** 1 Department of Plant Biotechnology, School of Biotechnology, Madurai Kamaraj University, Madurai, India; 2 Department of Life Sciences, CHRIST (Deemed to be University), Bengaluru, Karnataka, India

**Keywords:** chloroplast, nuclei, miRNA, photosystem II protein N, RACE-PCR

## Abstract

The microRNAs are endogenous, regulating gene expression either at the DNA or RNA level. Despite the availability of extensive studies on microRNA generation in plants, reports on their abundance, biogenesis, and consequent gene regulation in plant organelles remain naVve. Building on previous studies involving pre-miRNA sequencing in *Abelmoschus esculentus*, we demonstrated that three putative microRNAs were raised from the chloroplast genome. In the current study, we have characterized the genesis of these three microRNAs through a combination of bioinformatics and experimental approaches. The gene sequence for a miRNA, designated as AecpmiRNA1 (*A. esculentus* chloroplast miRNA), is potentially located in both the genomic DNA, i.e., nuclear and chloroplast genome. In contrast, the gene sequences for the other two miRNAs (AecpmiRNA2 and AecpmiRNA3) are exclusively present in the chloroplast genome. Target prediction revealed many potential mRNAs as targets for AecpmiRNAs. Further analysis using 5′ RACE-PCR determined the AecpmiRNA3 binding and cleavage site at the photosystem II protein N (psbN). These results indicate that AecpmiRNAs are generated from the chloroplast genome, possessing the potential to regulate mRNAs arising from chloroplast gene(s). On the other side, the possibility of nuclear genome-derived mRNA regulation by AecpmiRNAs cannot be ruled out.

## Introduction

MicroRNAs (miRNAs) are small noncoding RNAs (20–24 nt) involved in regulating gene expression through the degradation of target mRNA or by causing translation inhibition. Indeed, miRNAs play a crucial role in regulating various processes, including plant development, growth, molecular events, and responses to environmental stress conditions (Millar, 2020). These small RNA molecules are categorized as “intergenic and intronic” based on their location in the genome. Intergenic and intronic miRNAs in plants are transcribed with the assistance of DNA-dependent RNA Polymerase II (Pol II) in the nucleus. Intronic miRNAs are transcribed from spliced-out introns (Budak and Akpinar, 2015). The MIR gene (miRNA-encoding gene) is initially transcribed into a hairpin-like structure known as primary-miRNA (pri-miRNA) (Xie et al., 2005; Rogers and Chen, 2013). Subsequently, pri-miRNA is processed into miRNA by Dicer-like RNase III endonucleases (DCL), assisted by other accessory proteins (Fang and Spector, 2007; Dong et al., 2008).

Most studies on miRNA biogenesis have focused on the nuclear genome, leaving the presence of chloroplast miRNAs and their processing yet to be unambiguously determined. Conversely, a few studies have demonstrated the genesis of miRNAs from the mitochondrial genome (Vendramin et al., 2017; Fan et al., 2019).

Chloroplasts are semiautonomous organelles that carry out photosynthesis to produce energy-rich molecules required for plant growth. The chloroplasts sense the environmental stress conditions and produce many active compounds that help plants overcome various abiotic or biotic stress conditions (Zhang et al., 2019; Song et al., 2021). Retrograde signaling from chloroplast to the nucleus has been observed to favor miRNA biogenesis in the nucleus during plant stress (Crawford et al., 2018). During pathogen infection, the chloroplast becomes a primary target, damaging its functions and structure more than other plant cell organelles (Bhat et al., 2013; Bhattacharyya et al., 2015; Otulak et al., 2015; Srikakulam et al., 2022). Noncoding RNAs have been reported to regulate metabolic proteins in the chloroplast (Abdel-Ghany and Pilon, 2008; Anand and Pandi, 2021). Despite these studies, knowledge regarding miRNA biogenesis from chloroplasts is lacking, and the proposed origination in tomatoes remains ambiguous (Facella et al., 2017).

In 2017, we characterized miRNAs and their pre-cursors in the nonmodel plant *Abelmoschus esculentus* (Bhendi or Okra) through precursor miRNA sequencing (Kumar et al., 2017). Intriguingly, three precursor miRNAs showed possible origination from the chloroplast genome, raising questions about the potential role of miRNAs in regulating nuclear-derived or organelle mRNAs. In the current exploration, we determined the location of these miRNAs through computational observations such as Blast analysis and multiple sequence alignment, as well as experiments involving nuclei and chloroplast isolation, followed by PCR, RT-PCR, and qRT-PCR approaches. The results revealed that two miRNAs are possibly encoded by chloroplast DNA. The determination of the miRNA binding site by 5′ RACE-PCR showed that AecpmiRNA3 controls the photosystem II protein N (PsbN), affirming the role of organelle-originated miRNAs in the regulation of chloroplast mRNAs.

## Materials and methods

### Plants material collection and organelles isolation

*A. esculentus* plants, specifically the Arka anamika variety, were cultivated and maintained in a growth chamber with controlled conditions of 85% humidity and a temperature of 25˚C, following a 16/8 h day-night cycle. Leaves from plants aged 20–25 days were used for isolating chloroplasts and nuclei.

A protocol by Vieira et al. (2014) (*High salt plus saline Percoll gradient method* ) for chloroplast isolation was adapted with few modifications. Initially, freshly collected *A. esculentus* leaves were stored in the dark for 4–5 days at 4˚C to reduce the amount of starchy substances. Subsequently, 8–10 g of leaves were homogenized using an ice-cold isolation buffer (pH 3.8), comprising 1.25 M NaCl, 50 mM Tris-HCl (pH 8.0), 0.25 M ascorbic acid, 10 mM sodium metabisulfite, 7 mM EDTA, 0.0125 M Borax, 1% PVP-40 (w/v), and 0.1% BSA (w/v) in a prechilled mortar-pestle. The homogenate was filtered twice by using two layers of Miracloth (475855-1R, Millipore). Subsequently, the homogenate was loaded onto a Percoll gradient to achieve intact organelles. The subsequent centrifugation and pellet-washing steps for chloroplast isolation followed the procedure outlined by Vieira and coworkers (2014). A wash buffer (pH 8.0) containing 1.25 M NaCl, 50 mM Tris-HCl (pH 8.0), 7 mM EDTA, 0.0125 M Borax, 1% PVP-40 (w/v), and 0.1% BSA (w/v) was used for pellet washing and the same wash buffer was used for pellet suspension. The isolated chloroplasts were observed under both light and fluorescent microscopes and subsequently used for DNA isolation.

*A. esculentus* nuclei were isolated using the cellytic PN kit (CELLYTPN1, Sigma). *A. esculentus* leaves were stored in the dark at 4˚C for 4–5 days. A 6–7 g sample was finely minced in liquid nitrogen to form a powder, and the nuclei isolation buffer provided in the kit was added. Further filtration was conducted using Miracloth (475855-1R, Millipore). The filtrate was treated with 10% Triton X-100, with a 10-min incubation on ice to eliminate chloroplasts and cell membranes through lysis.

Following this, nuclei were isolated using a sucrose (1.7 M) density gradient. The lysate was applied on top of a 0.8 ml cushion of 1.7 M sucrose in 1.5 ml tubes (approximately 0.6 ml of lysate) and centrifuged at 12 000 × g for 10 min. The upper green phase and the sucrose cushion layer were aspirated without disturbing the pellet of nuclei. The pellet(s) was washed twice, by resuspending in 1 ml of the nuclei isolation buffer provided in the kit, followed by centrifugation for 5 min at 12 000 × g. Finally, the pellets were pooled from all the tubes.

Nuclei were stained using a propidium iodide solution (5mg/ml). A 5 μl propidium iodide solution was added to the nuclei suspension (10–20 μl), gently mixed by tapping, and incubated for 20–30 min at 4˚C. The stained nuclei were observed under a microscope.

### Total genomic DNA isolation

Genomic DNA from *A. esculentus* was isolated using a method adapted from Li et al. (2010) with some modifications. In brief, approximately 0.1 g of *A. esculentus* leaf sample was finely minced in liquid nitrogen to form a powder. One microliter of extraction buffer (10 mM Tris HCl, pH 7.5, 50 mM EDTA, 500 mM NaCl, 100 g/ml RNase A, 10 mM β mercaptoethanol) was added, gently mixed, and the homogenate was transferred to a microfuge tube with 20% SDS and mixed thoroughly. The mixture was incubated at 65˚C for 10 min. Following this, 500 μl of 5M potassium acetate was added, and the solution was incubated for 20 min on ice. The mixture was then centrifuged at 16 000 × g for 20 min at 4˚C. Subsequently, silica (100 mg/ml) was added to the supernatant, subjected to a 3–5 min incubation, and centrifuged at 16 000 × g for 30 s (4˚C). The pellet was washed with 70% ethanol, dried, and then resuspended in 40–50 μl of water, followed by incubation at 55˚C for 5 min. The supernatant was collected after centrifugation at 16 000 × g for 2 min and analyzed by agarose gel electrophoresis.

### Organelle DNA isolation

The isolation of chloroplast and nuclei DNA was performed using the plant genomic DNA isolation kit (DNeasy Plant Mini Kit, cat. nos. 69104, Qiagen) following the manufacturer’s instructions. Subsequently, the quality and quantity of the DNA were assessed using a NanoDrop ND-1000 spectrophotometer (Thermo Fisher Scientific, Waltham, MA, United States).

### Cloning

The DNA sequences of precursor molecules of AecpmiRNA1, AecpmiRNA2, and AecpmiRNA3 (hereafter referred to as Pre-AecpmiRNA) were amplified from chloroplast DNA. The amplicons of Pre-AecpmiRNA were observed through gel electrophoresis, and gel elution was carried out using the “silica method” (Boyle and Lew, 1995). Subsequently, ligation and transformation were performed using the cloning vector pTZ57 R/T (I PCR cloning kit, Thermo Scientific), and eluted PCR products were processed according to the protocol mentioned by Sambrook and Russell (2001). *Escherichia coli* cells containing recombinant plasmids were selected through blue-white screening, followed by plasmid extraction using the alkaline lysis method (Birnboim and Doly, 1979). Furthermore, pTZ57 R/T pre-AecpmiRNA1, pTZ57 R/T pre-AecpmiRNA2, and pTZ57 R/T preAecpmiRNA3 clones were validated by conducting restriction digestion with *Eco* R1, *Hind* III, *Xho* I, and *Bam* HI in accordance with the manufacturer’s protocol (ThermoFisher Scientific). The clones were subjected to sequencing to confirm the DNA sequences of preAecpmiRNAs, and nBLAST analysis was performed to verify their similarity with *A. esculentus* chloroplastoriginated pre-miRNAs.

### Total RNA isolation from A. esculentus

*A. esculentus* RNA was isolated using a modified CTAB method (Yang et al., 2008). In summary, 400 mg of leaves were finely minced into a powder in liquid nitrogen. Subsequently, 700–800 μl of extraction buffer I (2% CTAB (w/v), 0.1M Tris HCl (pH 8.0), 1.4 M NaCl, 20 mM EDTA (pH 8), 2% (w/v) PVP) and 700–800 μl of extraction buffer II (2% CTAB (w/v), 0.1 M Tris HCl (pH 8.0), 0.4 M NaCl, 20 mM EDTA (pH 8), 2% (w/v) PVPP), along with β-mercaptoethanol, were added before thawing. The tubes containing the homogenate were then incubated for 10 min at room temperature, followed by a brief vortexing. One microliter of chloroform was added, and the mixture was vortexed for 20 s, followed by centrifugation at 10 000 × g for 20 min at 4˚C. An equal volume of acid phenol:chloroform (1 : 1) was added to the aqueous phase, thoroughly mixed, and then centrifuged at 10 000 × g for 20 min at 4˚C. The top aqueous phase was transferred to a new tube, and an equal volume of chloroform:isoamyl alcohol (24 : 1) was added, mixed, and then centrifuged at 10 000 × g for 20 min at 4˚C. The resulting aqueous phase was transferred to a new tube, and 1/3 volume of LiCl (8 M) was added, mixed, and incubated for 4 h at −20˚C. After centrifugation at 10 000 × g for 20 min at 4˚C, the pellet was washed with 70% ethanol and then air-dried. Finally, the pellet was resuspended in RNase-free water. RNA yield and quality were assessed using a NanoDrop ND-1000 spectrophotometer (Thermo Fisher Scientific, Waltham, MA, United States) and agarose gel electrophoresis. MOPS (1.5%) buffer was used for gel preparation and electrophoresis due to its stable pH compared to other commonly used electrophoresis buffers.

### PCR

Pre-AecpmiRNA, 5.8S rRNA (nuclear gene-encoded), and rbcL (large subunit of ribulose-1,5-bisphosphate carboxylase, chloroplast genome-encoded protein) were amplified from the plant genomic DNA (20 ng) and chloroplast DNA (10–20 ng) using gene-specific primers (see Table 1). The amplification process involved an initial denaturation at 94˚C for 3 min, followed by 23 cycles of denaturation (94˚C for 30 s), annealing (60˚C for 1 min), and extension (72˚C for 30 s). The sequences of *A. esculentus* pre-miRNAs are listed in Table 2.

**Table 1:** List of the primers used for PCR, qRT-PCR, and 5′RLM-RACE PCR

Primer name	Sequence
rbcL forward	5′TTCCAAGCCGGTGTTAAAGAGT3′
rbcL reverse	5′CTCAGGCGGAACTCCAGGTT3′
5.8s forward	5′CAACGGATATCTCGGCTCTC3′
5.8s reverse	5′TTGCGTTCAAAGACTCGATG3′
Pre-AecpmiRNA1forward	5′GGCAATTACTCATTCTTAAAACCAGC3′
Pre-AecpmiRNA1 reverse	5′ATTCTTGTGGTTCCGGAGGATC3′
Pre-AecpmiRNA2 forward	5′GGATCCTCTTTTATGGTTCATATTCTGTG3′
Pre-AecpmiRNA2reverse	5′CTCGAGCCGTTGAGTTCTTACACTTCATG3′
Pre-AecpmiRNA3 forward	5′GGATCCAAACCAGATATAGAGATGGCGATA3′
Pre-AecpmiRNA3 reverse	5′CTCGAGTCATAGATCCATTGTGATCTA3′
qRT-PCR primers	
AecpmiRNA1_F	5′GGCGGGCGACCAAAGAGTC3′
^¶^ cDNAhp_1	5′GTCGTATCCAGTGCAGGGTCCGAGGTATTCGCACTGGATACGACCCCCCTCC3′
AecpmiRNA2_F	5′GCGGCGGTTCGTCCCTCT3′
^¶^ cDNAhp_2	5′GTCGTATCCAGTGCAGGGTCCGAGGTATTCGCACTGGATACGACACATATT3′
AecpmiRNA3_F	5′CGGCGTGCCCAGATATAGAG3′
^¶^ cDNAhp_3	5′GTCGTATCCAGTGCAGGGTCCGAGGTATTCGCACTGGATACGACGTCGCCA3′
Universal reverse primer	5′CCAGTGCAGGGTCCGAGGTA3′
YTH domain-containing protein	Forward 5′AAGAGCACCAACAGGAGGGTT3′ ^§^Reverse 5′AAGAGCACCAACAGGAGGGTT3′
e3 ubiquitin-protein ligase rglg1 isoform ×1( sacsin isoform ×3)	Forward 5′GAGGAATTACGATCAAGCTGCCC3′ Reverse 5′CAACGAGAAAACCCCTTCCTCAA3′
Methyl transferase nsun6 isoform	Forward 5′CCGAGGTCTCAAACCAAGAGCA3′ ^§^Reverse 5′TGACGTTAGGAAGACCATGCGA3′
alpha-xylosidase 1-like(PREDICTED: alpha-glucosidase)	Forward 5′CAACAGCTTTGCATTTTGGTAACCT3′ Reverse 5′CTTTTGCCCGTCAGATTCGCC3′
ethylene insensitive 3-like 3 protein isoform ×1	Forward 5′TTGATAAGAATGGGCTTGCTGCC3′ Reverse 5′AGACCGCTTTGCCGATTTCC3′
187-kDa microtubule-associated protein air9 isoform ×1	Forward 5′TGAAGGTGTTGTTATGTGTGGTCAG3′ Reverse 5′TTTCCTGTTTTTCCGTCGGCAC3′
psbN	Forward 5′TTTGGAATCATCTCTCTCCCGACT3′ ^§^Reverse 5′CCGGATTTGGATGCTCAAGTGG3′
3-ketoacyl-CoA thiolase 5	Forward 5′TCCGTCTTTGGGTTAACTTTGTTGA3′ ^§^Reverse 5′GCAACCGAGTGTAGGATGGC3′
splicing factor 3b subunit 3-like	Forward 5′GCTGGCCATTATAGCACTGTGT3′ ^§^Reverse 5′GCCATGAGCATCAGAAAGGGTT3′
Primers for 5′RLM RACE-PCR	
Methyl transferase nsun6 isoform	ORP 5′TGACGTTAGGAAGACCATGCGA3′
psbN	ORP 5′CCGGATTTGGATGCTCAAGTGG3′
3-ketoacyl-CoA thiolase 5	ORP 5′GCAACCGAGTGTAGGATGGC3′

¶ hairpin loop primers used for cDNA conversion for miRNAs;

§ the reverse primers used for RT-PCR were gene-specific inner reverse primers for 5′RLM RACE-PCR; ORP – outer reverse primer

**Table 2:** Sequence of precursor *A. esculentus* microRNAs and mature microRNAs

Name	Sequence (5′-3′)
Pre-AecpmiRNA1	ACCAGCATTCTTAAGACCAAAGAGTCGGAGGGGGGAAAGCTCTCCGTTCCTGGTTCTCCTGTAGC TGGA
Pre-AecpmiRNA2	TCTTTTATGGTTCATATTCTGGATTGGGTTCGTCCCTCTAATATGTAATATAAGATGTAATAATGG GATGAACTAAGTTATAGGCATGAAAGTGTAAGAACTCAACGG
Pre-AecpmiRNA3	AAACCAGATATAGAGATGGCGACTAAGGTTGCTGTTTCCATTATTATATAATTTCAAGATCACAAT GGATCTATGA
AecpmiRNA1	GACCAAAGAGTCGGAGGGGG
AecpmiRNA2	TTCGTCCCTCTAATATGT
AecpmiRNA3	CCAGATATAGAGATGGCGAC

### RT-PCR

*A. esculentus* total RNA sample (10 μg) was treated with 3U Turbo DNase (2 U/μl, Ambion, Austin, Texas, United States) at 37˚C for 30–40 min to remove DNA from the RNA sample. The reaction was subsequently heat-inactivated using 0.01 mM EDTA for 10 min at 72˚C to deactivate the DNase. The quantity and quality of the RNA were assessed using NanoDrop ND-1000 spectrophotometer readings (Thermo Fisher Scientific, Waltham, MA, United States) and by performing 1.5% MOPS agarose gel electrophoresis. Next, complementary DNA was synthesized from 500 ng RNA using the RevertAid First Strand cDNA Synthesis Kit (ThermoFisher Scientific) following the manufacturer’s protocol. Random hexamer primers provided in the cDNA synthesis kit were employed for the reverse transcription reaction. The annealing temperature for RT-PCR primers (see Table 1) was optimized to obtain a specific band for the target DNA. RT-PCR was carried out using 15–20 ng of cDNA, following standardized PCR conditions. Water and RNA were utilized as negative controls during RT-PCR.

### Stem-loop RT-PCR

The stem-loop RT-PCR method was adopted to assess the expression of AecpmiRNA1, AecpmiRNA2, and AecpmiRNA3. MiRNA-specific hairpin primers were used for the reverse transcription reaction (Table 1). As a result, there were four different setups for the reverse transcription reaction, including the internal control gene, U6snRNA. In the reverse transcription reaction, 300 ng of DNase-treated RNA sample was combined with miRNA-specific hairpin primer (20 pmol) and incubated for 5 min at 70˚C. The incubated sample was then mixed with 5 × reaction buffer, 10 μM dNTP, ribolock RNase inhibitor, 0.4 μl RevertAid RT (200 U/μl), and nuclease-free water (Ambion, Thermo Scientific, Waltham, MA, United States). This mixture was incubated at 42˚C for 1 h. As a negative control, another reaction was set up with all the components, where water was added instead of RNA. The method for designing hairpin primers was adapted from Adhikari et al. (2013). Stemloop RT-PCR was performed following the protocol outlined by Adhikari et al. (2013). *A. esculentus* AecpmiRNAs sequences are provided in Table 2.

### qRT-PCR

qRT-PCR was performed using gene-specific primers to assess the relative transcriptional expression of the miRNAs and their respective target mRNAs. U6 snRNA and 5.8s rRNA were employed as internal controls for miRNAs and target transcripts, respectively. In each 20 μl reaction, 20 ng of cDNA and 2 pmol of each primer were used. Amplification was monitored using the fluorescent dye SYBR® Green (Roche Diagnostics, GmBH, Germany) in a QuantStudio™ 1 real-time PCR system (Thermo Scientific, Waltham, MA, United States). qRTPCR was performed with three biological and experimental repetitions. The relative changes in the expression of miRNAs and targets with respect to the internal reference gene were analyzed using the 2^−ΔΔCT^ method (Livak and Schmittgen, 2001). Specifically, ΔCT was calculated by subtracting the CT mean reference (internal control) from the CT mean target. Subsequently, ΔΔCT values were calculated by subtracting the lowest CT value from triplicates of the internal control sample from the ΔCT of the target sample. Finally, the fold difference was calculated using 2^−ΔΔCT^.

### 5′RLM-RACE PCR for target validation

The FirstChoice® RLM-RACE Kit (Thermo Scientific, Waltham, MA, United States) was employed for the 5′ end amplification of target cDNA, following the manufacturer’s instructions with some modifications, specifically excluding calf intestine alkaline phosphatase (CIP) and tobacco acid pyrophosphatase (TAP) treatments. *A. esculentus* RNA (1 μg) was ligated with a 5′RACE adaptor, and the cDNA strand was synthesized using 2 μl of adaptor-ligated RNA. Subsequently, nested PCR was conducted using gene-specific outer and inner reverse primers and 5′ adaptor primers (Table 1). RACE PCR products were analyzed by running 2% agarose gel electrophoresis, and DNA bands of the expected size were eluted and cloned into the pJET Blunt 1.2 vector (CloneJET PCR Cloning Kit—Thermo Fisher Scientific) following the protocol outlined by Sambrook and Russell (2001). The cloned plasmid containing the RACE PCR product was subjected to restriction digestion using *Bgl* II. The restriction digestion reaction involved 0.5–0.6 μg of DNA in a 20 μl reaction volume with 1 μl of the restriction enzyme. Finally, the cloned plasmid harboring the RACE PCR product was sequenced using Sanger sequencing.

### Computational analysis

Nucleotide BLAST analysis was conducted to verify the existence of DNA sequences encoding all three Pre-AecpmiRNAs. In this analysis, the nucleotide sequence of each pre-miRNA was entered as a query sequence, and the megablast program or high-similarity sequence was selected under the program selection section. The standard database was chosen, with a specific focus on *Malvaceae* in the organism section.

Additionally, Nucleotide BLAST analysis was also carried out for the cloned sequences of Pre-AecpmiRNAs.

Multiple sequence alignment for Pre-AecpmiRNAs was executed by selecting 5–6 chloroplast genome sequences from different plants in the *Malvaceae* family. The Clustal W tool was utilized for alignment analysis. Rather than entering the entire genome, only the portion showing similarity with Pre-AecpmiRNAs, along with extra flanking nucleotides, was selected for the alignment study.

To predict potential mRNA targets for AecpmiRNAs, an online server named psRNATARGET was used. *A. esculentus* mRNA sequencing data, generated in our lab, was used as the source of mRNAs. AecpmiRNA sequences were submitted to psRNATARGET to identify potential targets.

## Results

### miRNAs origin confirmation

A detailed analysis of *A. esculentus* pre-miRNA sequencing data by Kumar et al., (2017), utilizing in-house assembled tools, unveiled novel miRNAs with precursor sequences mapped to the chloroplast genome. Interestingly, the existence, biogenesis, and regulation of chloroplast miRNAs are currently ambiguous, with a single report speculating on the potential origin of miRNAs from tomato chloroplasts (Facella et al., 2017). In order to substantiate the chloroplast origin of miRNAs from *A. esculentus*, the pre-miRNA sequences obtained from the sequencing study (Kumar et al., 2017) were retrieved, and *in silico* approaches were performed.

**Fig. 1:**
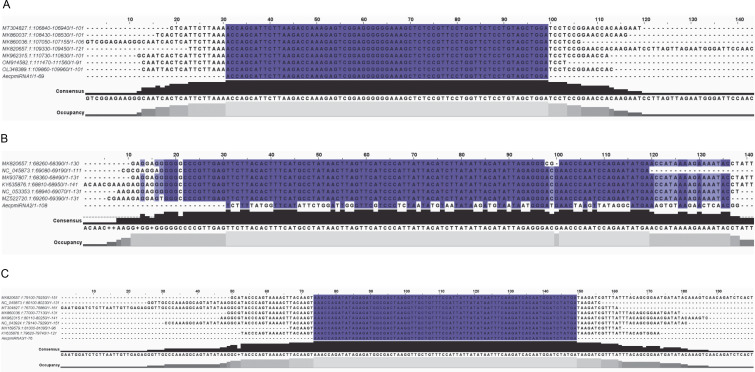
Multiple sequence alignment of Pre-AecpmiRNAs confirmed the presence of pre-AecpmiRNAs sequences in the chloroplast genome; sequence alignment of (A) Pre-AecpmiRNA1, (B) Pre-AecpmiRNA2, and (C) Pre-AecpmiRNA3 sequences with chloroplast DNA from various *Malvaceae* family plants was performed

Blast analysis showed that these sequences aligned well with the chloroplast genome of the *Malvaceae* family. To further confirm that these three pre-miRNAs are primarily present in the *Malvaceae* chloroplast genome, multiple sequence alignment was conducted using chloroplast genomes from various *Malvaceae* family plants along with precursor miRNA sequences. All three *A. esculentus* Pre-AecpmiRNAs were observed to be present across the *Malvaceae* family (Fig. 1).

**Fig. 2:**
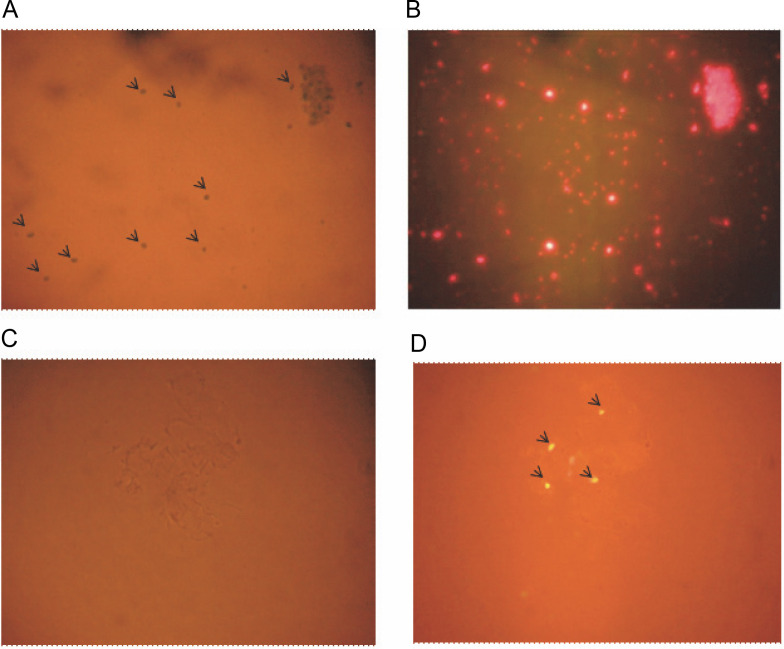
Microscopic observation of isolated *A*. *esculentus* chloroplasts and nuclei; image of chloroplasts under (A) light microscope and (B) fluorescent microscope; image of Propidium iodide stained nuclei under (C) light microscope and (D) fluorescent microscope; arrows show the isolated chloroplasts (A) and nuclei (D)

Bioinformatics analysis prompted us to validate the presence and location of these miRNAs through experimental methods. Initially, PCR was performed with total DNA isolated from *A. esculentus* leaves as the template for all three pre-miRNA primers, showing good amplification and confirming the presence of these miRNAs in *A. esculentus* (Supplementary Fig. S1). To gain a clearer understanding of whether these pre-miRNAs are located in the chloroplasts, nuclear genome, or both, we optimized a protocol to isolate chloroplasts and nuclei, as described in the Materials and Methods section. *A. esculentus* chloroplasts were examined under the light and fluorescent microscope which demonstrated the successful isolation of chloroplasts (Fig. 2A, Fig. 2B). Fluorescent microscopic observation of isolated *A. esculentus* nuclei stained with propidium iodide ensured their proper isolation (Fig. 2C, Fig. 2D).

**Fig. 3:**
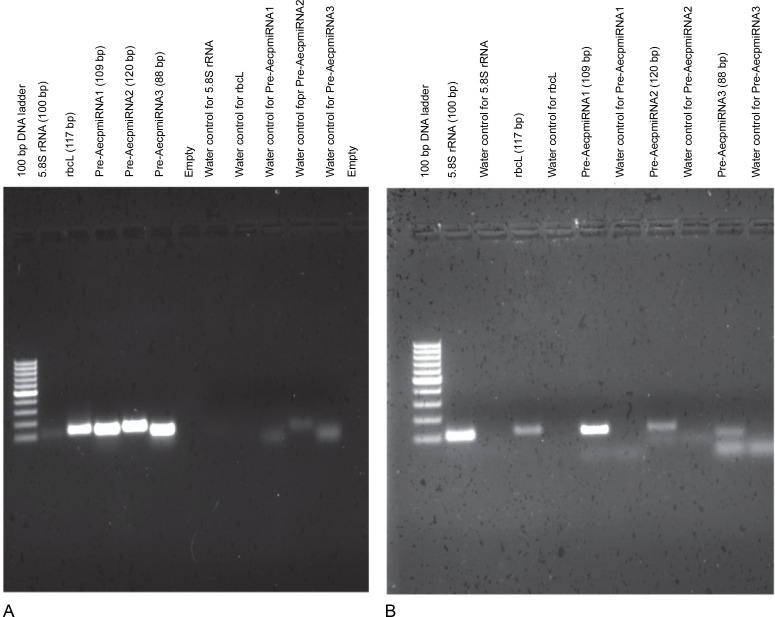
PCR reaction confirmed the chloroplast origin of *A. esculentus* Pre-AecpmiRNA1, Pre-AecpmiRNA2, and Pre-AecpmiRNA3; *A. esculentus* chloroplast and nuclei DNA, obtained from three different isolations, were used as templates; Figures 3A, 3B shows gel images of PCR performed with cpDNA (A) and nuclei DNA (B) for pre-AecpmiRNAs along with 5.8S rRNA and rbcL genes; the same experiment was done by using three biological replicates; the remaining gel pictures have been provided as supplementary Figure S2

To confirm the chloroplast origin of Pre-AecpmiRNA1, Pre-AecpmiRNA2, and Pre-AecpmiRNA3, PCR was carried out with *A. esculentus* chloroplast or nuclei DNA (Fig. 3A, Fig. 3B, and Supplementary Fig. S2). When PCR was performed with chloroplast genomic DNA, the amplification of the three precursor microRNAs and the chloroplast DNA-encoded rbcL gene was nearly equivalent (Fig. 3A). Conversely, the amplification for the nuclear DNA-encoded 5.8S rRNA gene was comparatively lower. Thus, the results of PCR with cpDNA (chloroplast DNA) template suggest that transcripts for AecpmiRNA1, AecpmiRNA2, and AecpmiRNA3 are likely present in the chloroplast genome.

The PCR results using nuclear DNA as the template exhibited robust amplification for the 5.8S rRNA gene compared to the chloroplast DNA-encoded rbcL gene, indicating a relatively pure and less cpDNA-contaminated nuclear DNA sample. In the case of nuclear DNA, the PCR results showed reduced amplification of rbcL, Pre-AecpmiRNA2, and Pre-AecpmiRNA3 genes, while the Pre-AecpmiRNA1 gene showed good amplification (Fig. 3B). Intriguingly, the successful amplification of the Pre-AecpmiRNA1 DNA sequence from A. esculentus nuclear DNA suggests the possible presence of the PreAecpmiRNA1 sequence in the nuclear genome.

To substantiate the computational and PCR-based findings, Pre-AecpmiRNAs were amplified from chloroplast DNA and subsequently cloned for further study (Supplementary Fig. S3). The sequencing results for each cloned DNA sequence of precursor miRNAs (Supplementary file) underwent an nBLAST search, revealing 100% similarity with the DNA sequence of Pre-Aecpmi RNAs existing in the *A. esculentus* chloroplast genome database. Consequently, the sequencing of cloned DNA sequences of the precursor miRNAs provided compelling evidence supporting the chloroplast origin of *A. esculentus* Pre-AecpmiRNA1, Pre-AecpmiRNA2, and PreAecpmiRNA3.

### Target prediction and qRT-PCR to quantify miRNA and their targets

In our analysis, we utilized the *A. esculentus* transcriptome generated in our lab (Priyavathi et al., 2018) and the transcriptome provided by the Roland lab (Schafleitner et al., 2013) from “The World Vegetable Center, Taiwan” to identify targets for *A. esculentus* miRNAs through the online server psRNA Target (Dai and Zhao, 2011). Using *A. esculentus* transcriptome sequences along with AecpmiRNA1, AecpmiRNA2, and AecpmiRNA3 sequences as input, many targets were predicted. From the predicted target list, we selected targets based on the expected value scores (Supplementary Table S1). After identifying potential targets for AecpmiRNA1, AecpmiRNA2, and AecpmiRNA3, we intended to check their expression profile of miRNAs and their respective targets as well. However, before proceeding with expression profiling, stem-loop RT-PCR for miRNAs (Supplementary Fig. S4) and RT-PCR for their respective targets (Supplementary Fig. S5) was carried out to confirm their expression and to prevent any non-specific amplification during qRT-PCR. The expression of AecpmiRNAs in leaves was confirmed through stemloop RT-PCR (Supplementary Fig. S4), with optimized annealing temperatures to obtain the expected products.

**Fig. 4:**
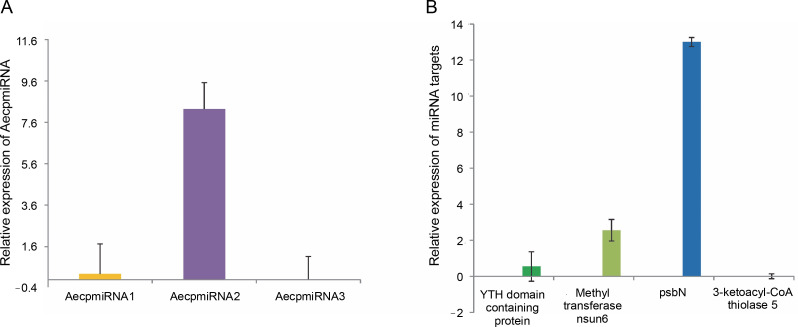
Expression pattern of AecpmiRNAs and their targets; (A) relative expression of AecpmiRNA1, AecpmiRNA2, and AecpmiRNA3 was analyzed by performing qRT-PCR; (B) Relative expression of mRNA targets of AecpmiRNA1 (YTH domain-containing family protein 2-like isoform and methyltransferase nsun6 isoform) and AecpmiRNA3 (psbN and 3-ketoacyl-CoA thiolase 5) was analyzed by performing qRT-PCR; error bars indicate ±SE of three biological replicates

**Fig. 5:**
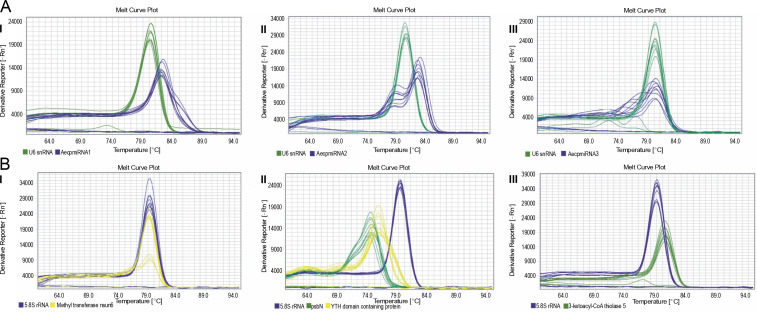
Dissociation curve for qRT-PCR performed for AecpmiRNAs and their targets; (A) shows the melt curve analysis for AecpmiRNA1 (AI), AecpmiRNA2 (AII), and AecpmiRNA3 (AIII); (B) shows the melt curve analysis for mRNA targets methyltransferase nsun6 isoform (BI), psbN, and Yth domain-containing family protein 2-like isoform (BII) and 3-ketoacyl-CoA thiolase 5(BIII)

Upon confirming that the primers amplified only the expected products for miRNAs and their targets (Supplementary Fig. S4 and Fig. S5), qRT-PCR was performed to obtain the expression profiles for AecpmiRNAs and their target mRNAs (Fig. 4A, Fig. 4B). The expression profile analysis of AecpmiRNAs demonstrated that the expression of AecpmiRNA3 was the lowest among all three miRNAs, whereas AecpmiRNA2 showed a higher level of expression (Fig. 4A). AecpmiRNA2 expression was more than 29 and 500-fold higher than AecpmiRNA1 and AecpmiRNA3, respectively. Regarding the target sequences, the expression profile analysis showed that the expression level of methyltransferase nsun6 isoform was higher (2^−ΔΔCt^ is 2.558) than the respective AecpmiRNA1 expression (2^−ΔΔCt^ is 0.014). The relative fold change in the expression level of psbN (2^−ΔΔCt^ is 13.005) was higher compared to the expression level of its respective AecpmiRNA3 (2^−ΔΔCt^ is 0.278), while another target of AecpmiRNA3, 3-ketoacyl-CoA thiolase 5, showed lower expression (2^−ΔΔCt^ is 0.000671). The target mRNAs of AecpmiRNA2 either showed no amplification or nonspecific amplification in RT-PCR. Dissociation curve analysis for cDNAs of miRNA and targets demonstrated the specificity of our qRT-PCR assay (Fig. 5).

### miRNA target validation and identification of miRNA pairing site

**Fig. 6:**
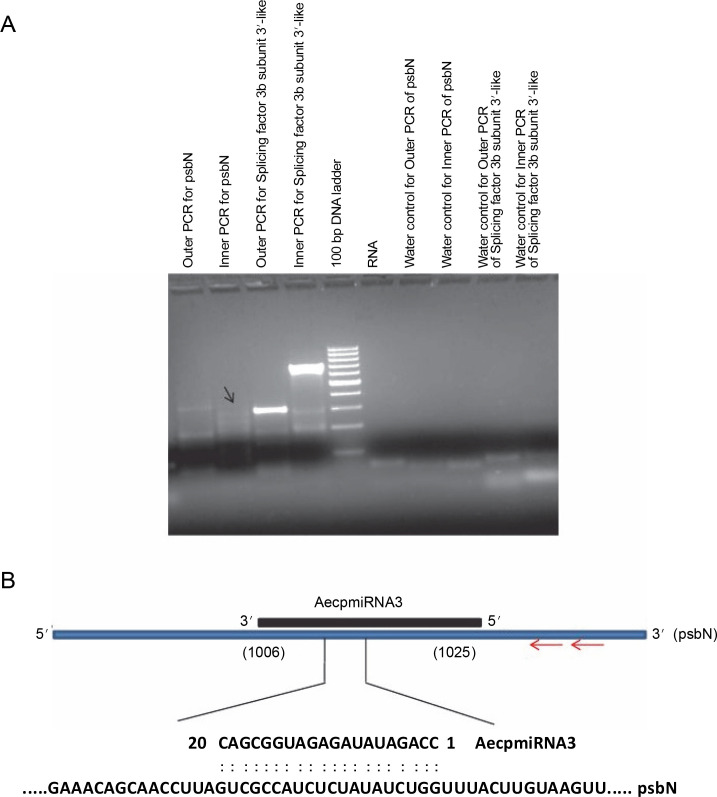
RACE PCR for AecpmiRNA3 targets and schematic representation of AecpmiRNA3 induced cleavage site in psbN transcript; (A) the arrow indicates the PCR band cloned and sequenced to analyze the AecpmiRNA3 cleavage site in the psbN transcript; RACE PCR for another AecpmiRNA3 target (splicing factor 3b subunit 3-like) gave nonspecific amplification; RNA and water were used as negative controls; (B) the red arrow shows the gene-specific primer sites for 5′RLM-RACE PCR; the numbers in brackets show the position of nucleotide in target mRNA which is complementary to AecpmiRNA3

To validate the results obtained from the psRNA Target, 5′ RLM-RACE experiments were conducted for miRNA targets whose expression patterns showed a noticeable correlation with their respective miRNA expressions (Fig. 4) (Supplementary Table S1). Specifically, methyltransferase nsun6 isoform (target for AecpmiRNA1) and psbN (target for AecpmiRNA3) were chosen for 5′ RLM-RACE analysis. For the 5′ RLMRACE experiments, *A. esculentus* cDNA, synthesized by reverse transcription of adaptor-ligated RNA, was used. Gene-specific outer reverse and inner reverse RACE primers (Table 1) were used for RACE-PCR. Unfortunately, the methyltransferase nsun6 isoform as the target for AecpmiRNA1 could not be validated due to a nonspecific RACE product. However, the RACE-PCR product for psbN precisely matched with the target sequence (psbN), indicating AecpmiRNA3-mediated regulation of photosynthesis-related genes (Fig. 6A, Fig. 6B).

## Discussion

In general, miRNAs are generated from the nuclear genome but exceptionally, few reports have demonstrated their location inside the organelles (Barrey et al., 2011; Ro et al., 2013). Similar to these observations, small RNA and their precursor RNAs profiling from *A. esculentus* by Kumar et al. (2017) have identified three novel microRNAs named AecpmiRNA1, AecpmiRNA2, and AecpmiRNA3 and predicted their probable origin from the chloroplast genome. In the present study, experimental validations were carried out to examine the NGS data-based predictions on *A. esculentus* AecpmiRNA1, AecpmiRNA2, and AecpmiRNA3. With the combinations of computational analysis, cloning, and reliable stem-loop RT-PCR, we demonstrated that these miRNAs are certainly present in the chloroplast. Out of three miRNAs, AecpmiRNA1 is probably present also in the nuclear genome. To date, there are no proper reports on microRNA generation from the chloroplast genome. In contrast, in the case of mitochondria (human), there are many studies explaining their presence in mitochondria either coded by nuclear DNA or mitochondrial DNA (Barrey et al., 2011; Vendramin, 2017; Rodrigues et al., 2020). Intriguingly, colocalization of miRNA-365, pre-mir-302a, and pre-let-7b has been documented by *in situ* hybridization in mitochondria of human myoblasts (Barrey et al., 2011). In their study, Barrey et al. (2011) speculated that either miRNA should be imported from cytosol or be partially processed inside mitochondria. The presence of precursor miRNA sequences in organellar DNA indicates that the organelle might possess miRNA biogenesis machinery and RNA interference complex. On the other side, it can be speculated that precursor sequences might be transported to the cytosol for their processing similarly to nuclear-encoded miRNAs. Interestingly, one of the important RNA interference protein components “Ago2” has been colocalized with mitochondria (Bandiera et al., 2011; Zhang et al., 2014). Additionally, Wang and coworkers (2015) found Dicer and AGO-associated miRNA in mitochondria fraction suggesting the presence of a typical processing pathway and RNAi complex in mitochondria. In another study by Kretov et al. (2020), it was seen that an erythrocyte miRNA “miR-451” is dependent on Ago2 for processing rather than Dicer. Hence, similar to erythrocyte miRNAs, there could be a possibility that organellar miRNAs may be following a non-canonical pathway for their biogenesis.

However, there is no reported presence of Dicer-like proteins or Ago in the chloroplast. Instead, other RNAbinding proteins with exonuclease and endonuclease activities have been proposed to be involved in organellar RNA processing and the generation of small noncoding RNA molecules (Anand and Pandi, 2021). The study suggests that similar to mitochondrial small RNAs, pre-miRNAs may be transported into the cytosol for processing, or there may be an RNAi complex within chloroplasts for miRNA maturation.

The expression analysis of *A. esculentus* psbN indicated a higher level of this transcript compared to its respective microRNA, AecpmiRNA3, suggesting a negative correlation between AecpmiRNA3 and psbN transcript levels. This led to the speculation that AecpmiRNA3 might be regulating psbN expression. The psbN gene is localized in stroma lamellae and is required for photosystem II assembly (Torabi et al., 2014). Transcription of the psbN gene is known to be regulated by sigma 3 (transcription factor) and plastid-encoded polymerase (PEP) (Zghidi et al., 2007), while post-transcriptional regulation of psbN has not been observed. Additionally, the translation of psbN is controlled by a pentatricopeptide repeat protein (PPR) named “LEP1” (Williams-Carrier et al., 2019).

The observation of an AecpmiRNA3-induced cleavage site in the psbN transcript through 5′RLM-RACE PCR is significant. Considering that psbN is transcribed from cpDNA, this observation suggests that chloroplasts could regulate psbN mRNA expression through organellar miRNA.

Photosynthesis is a stress-sensitive process, and alterations in the expression of proteins related to photosynthesis have been observed under stress conditions (Li et al., 2017; Zhang et al., 2017). While there are reports describing the roles of miRNAs in the direct or indirect regulation of photosynthesis (Abdel-Ghany and Pilon, 2008; Zhang et al., 2017; Kajal et al., 2019), the detailed study of AecpmiRNA3 and psbN expression under various conditions may reveal additional regulatory pathways for genes encoding photosynthesis-related proteins.

## Conclusion

The findings from this study provide evidence for the existence of miRNAs generated from the chloroplast genome. Specifically, the precursor sequences of two miRNAs, Pre-AecpmiRNA2 and Pre-AecpmiRNA3, were identified as originating from chloroplast DNA, while pre-AecpmiRNA1 is suggested to be present in both the nuclear and chloroplast genomes. Traditionally, organelle gene expression in chloroplasts has been thought to be regulated by RNA-binding proteins. However, the study’s results, including the expression analysis of AecpmiRNAs and their targets, challenge this notion and suggest that small noncoding RNAs may also play roles in post-transcriptional gene regulation within the chloroplast. The study further highlights the potential of these organellar miRNAs to regulate important nuclear-encoded mRNAs. Given that the chloroplast is a primary target for plant pathogens, understanding the expression status of these chloroplast miRNAs and their related target transcripts becomes crucial. This knowledge could contribute to uncovering key molecules involved in suppressing pathogenic invasion during biotic stress and responding to various abiotic stress conditions. Future gene silencing studies on AecpmiRNAs may provide insights into their importance in maintaining chloroplast homeostasis.

## Supplementary Material

Evidence of microRNAs origination from chloroplast genome and their role in regulating Photosystem II protein N (psbN) mRNA
