# Supplementary material for: Evidence of microRNAs origination from chloroplast genome and their role in regulating Photosystem II protein N (psbN) mRNA
Source: BioTechnologia (Pozn). 2024 Mar 29;105(1):19–32. doi: 10.5114/bta.2024.135639 (PMC11020153; doi:10.5114/bta.2024.135639)
Supplement: Evidence of microRNAs origination from chloroplast genome and their role in regulating Photosystem II protein N (psbN) mRNA [file BTA-105-1-52454-s001.pdf]

BioTechnologia vol. 105 (1) • 2024
Journal of Biotechnology, Computational Biology and Bionanotechnology SUPPLEMENTARY MATERIALS
https://doi.org/10.5114/bta.2024.135639

CC BY 4.0

Evidence of microRNAs origination from chloroplast genome and their role in regulating Photosystem II protein N (psbN) mRNA

Asha Anand [1,2]*, Shailja Chauhan [1], Aparna Chodon [1], Kavitha Velayudha Vimala Kumar [1], Saravanakumar S. [1], Gopal Pandi [1]

[1] Department of Plant Biotechnology, School of Biotechnology, Madurai Kamaraj University, Madurai, India

[2] Department of Life Sciences, CHRIST (Deemed to be University), Bengaluru, Karnataka, India

Received: 11 June 2023; revised: 25 December 2023; accepted: 5 January 2024

Abstract

The microRNAs are endogenous, regulating gene expression either at the DNA or RNA level. Despite the availability of extensive studies on microRNA generation in plants, reports on their abundance, biogenesis, and consequent gene regulation in plant organelles remain naïve. Building on previous studies involving pre-miRNA sequencing in *Abelmoschus esculentus*, we demonstrated that three putative microRNAs were raised from the chloroplast genome. In the current study, we have characterized the genesis of these three microRNAs through a combination of bioinformatics and experimental approaches. The gene sequence for a miRNA, designated as AecpmiRNA1 (*A. esculentus* chloroplast miRNA), is potentially located in both the genomic DNA, i.e., nuclear and chloroplast genome. In contrast, the gene sequences for the other two miRNAs (AecpmiRNA2 and AecpmiRNA3) are exclusively present in the chloroplast genome. Target prediction revealed many potential mRNAs as targets for AecpmiRNAs. Further analysis using 5′ RACE-PCR determined the AecpmiRNA3 binding and cleavage site at the photosystem II protein N (psbN). These results indicate that AecpmiRNAs are generated from the chloroplast genome, possessing the potential to regulate mRNAs arising from chloroplast gene(s). On the other side, the possibility of nuclear genome-derived mRNA regulation by AecpmiRNAs cannot be ruled out.

Key words: chloroplast, nuclei, miRNA, photosystem II protein N, RACE-PCR

Table S1. List of the mRNA targets obtained through an online tool psRNA target

miRNA	Target mRNA	Functional importance
AecpmiRNA1	Yth domain-containing family protein 2-like isoform × 1	specifically recognizes and binds N6-methyladenosine (m6A)-containing RNAs, and regulates mRNA stability
	e3 ubiquitin-protein ligase rglg1 isoform × 1 (sacsin isoform × 3)	abscisic acid signaling pathway
	methyltransferase nsun6 isoform × 1	RNA binding methyltransferase activity
AecpmiRNA2	alpha-xylosidase 1-like (PREDICTED: alpha-glucosidase)	hydrolase activity, hydrolyze O-glycosyl compounds
	187-kda microtubule-associated protein air9 isoform × 1	involved in maturation of cell plates
	ethylene insensitive 3-like 3 protein isoform × 1	transcription factor activity, sequence-specific DNA binding
AecpmiRNA3	PsbN (Photosystem II reaction center protein N)	involved in assembly of photosystem II reaction center
	3-ketoacyl-CoA thiolase 5	probably involved in long chain fatty-acid beta-oxidation prior to gluconeogenesis during germination and subsequent seedling growth
	splicing factor 3b subunit 3-like	involved in RNA splicing

* Corresponding author: [1] Department of Plant Biotechnology, School of Biotechnology, Madurai Kamaraj University, Madurai, India; [2] Department of Life Sciences, CHRIST (Deemed to be University), Bengaluru, Karnataka, India; e-mail: ashaanand@mkuniversity.org; asha.anand@christuniversity.in

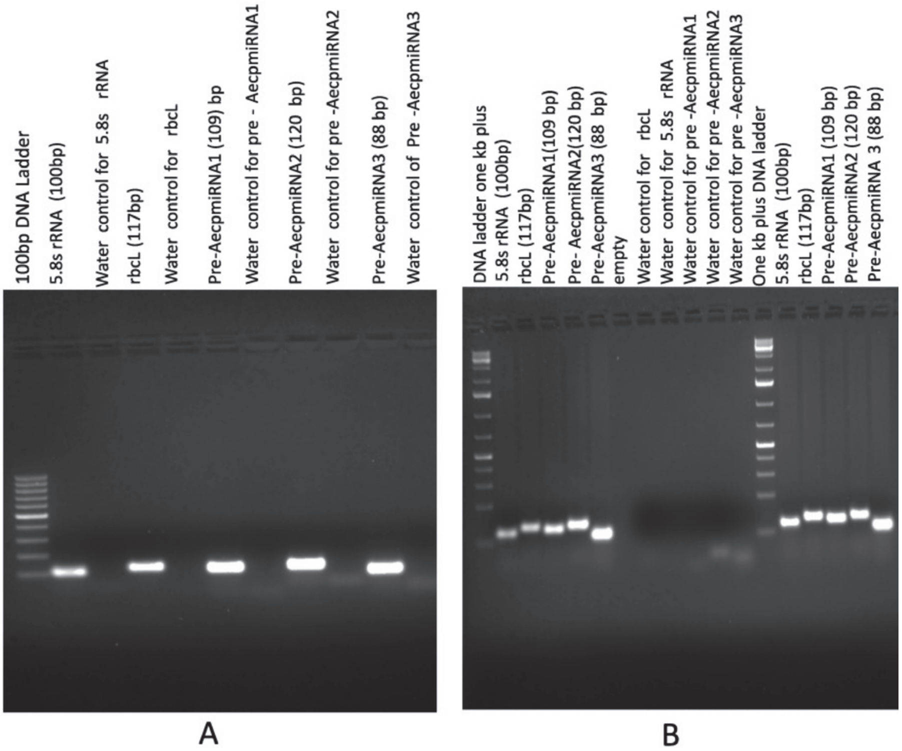

Fig. S1. PCR for Pre-AecpmiRNA1, Pre-AecpmiRNA2 and Pre-AecpmiRNA3 by using *A. esculentus* genomic DNA as template; A and B are gel images of PCR done with DNA obtained from three biological replicates

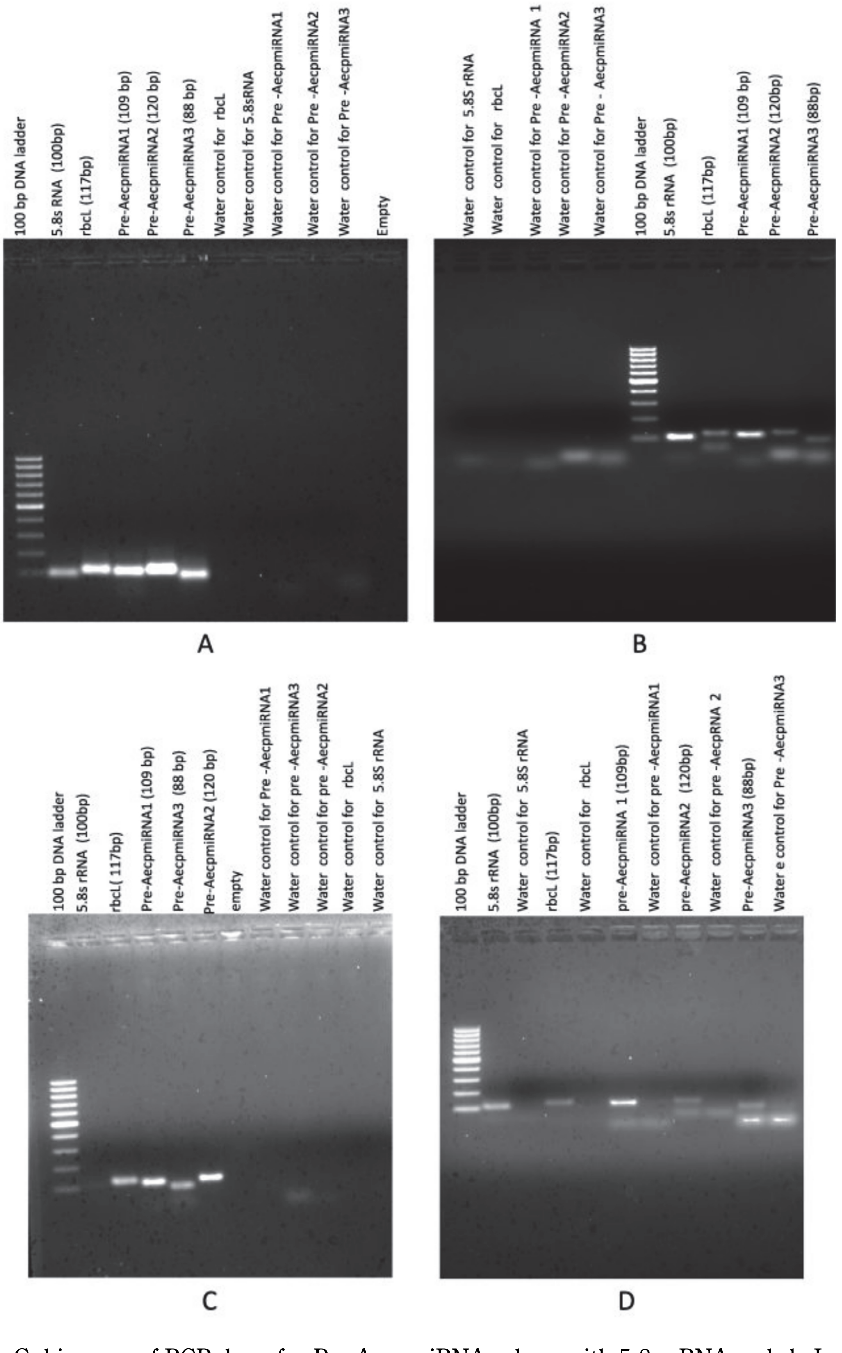

Fig. S2. Gel images of PCR done for Pre-AecpmiRNAs along with 5.8s rRNA and rbcL genes;
PCR was carried out using (A, C) *A. esculentus* cpDNA and (B, D) nuclei DNA from two biological replicates

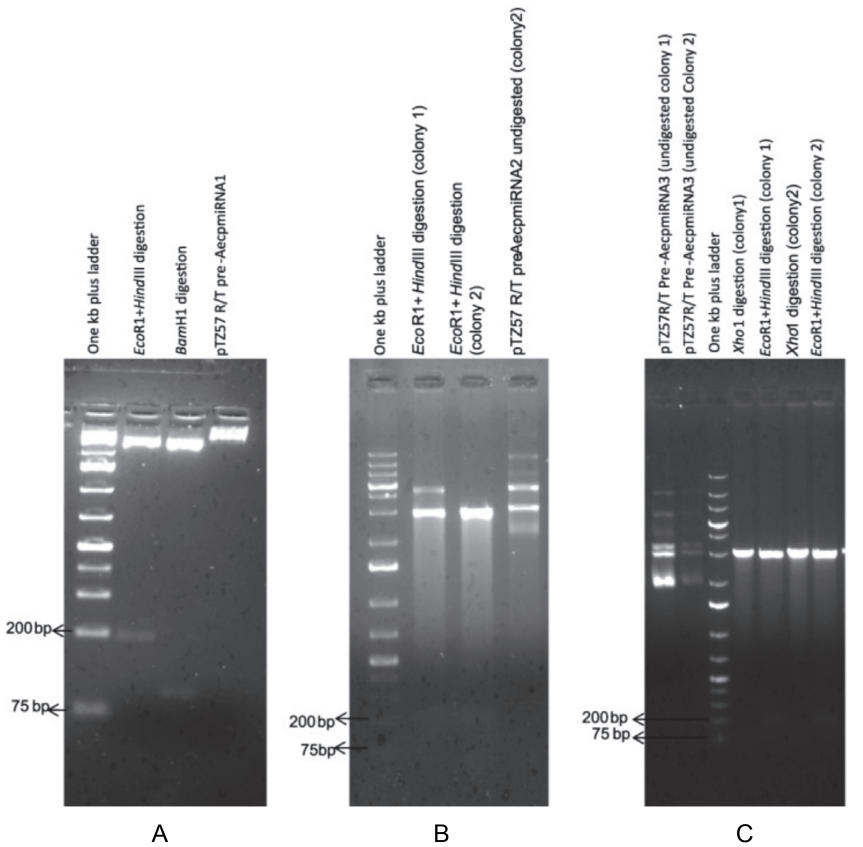

Fig. S3. Confirmation of cloning of Pre-AecpmiRNAs in pTZ57R/T vector through restriction digestion using appropriate enzymes and sequencing; EcoR1 and HindIII sites are present in the vector adjacent to the cloning site; thus, double digestion with these enzymes showed a release of DNA fragments of the expected size in all clones; A) restriction digestion of pTZ57R/T Pre-AecpmiRNA1; BamHI is present in the vector and pre-miRNA as well; B) restriction digestion of pTZ57R/T Pre-AecpmiRNA2; C) restriction digestion of pTZ57R_T Pre-AecpmiRNA3; Xho1 is the unique restriction site present in Pre-AecpmiRNA3

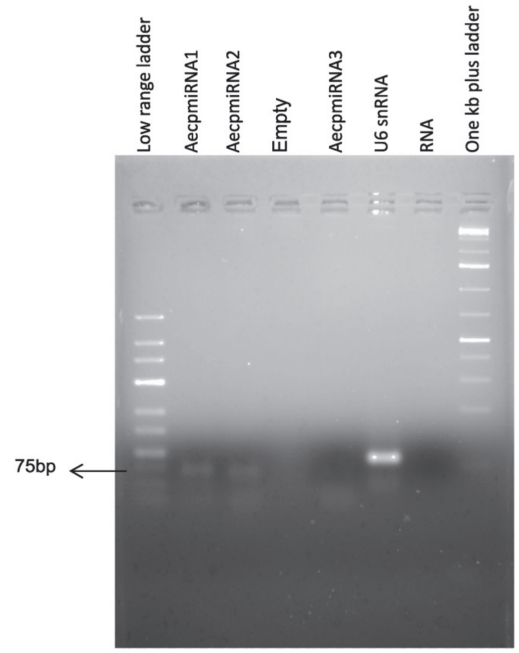

Fig. S4. Stem-loop RT-PCR for *A. esculentus* AecpmiRNA1, AecpmiRNA2 and AecpmiRNA3; U6 snRNA was used as internal control; RNA was taken as negative control for PCR

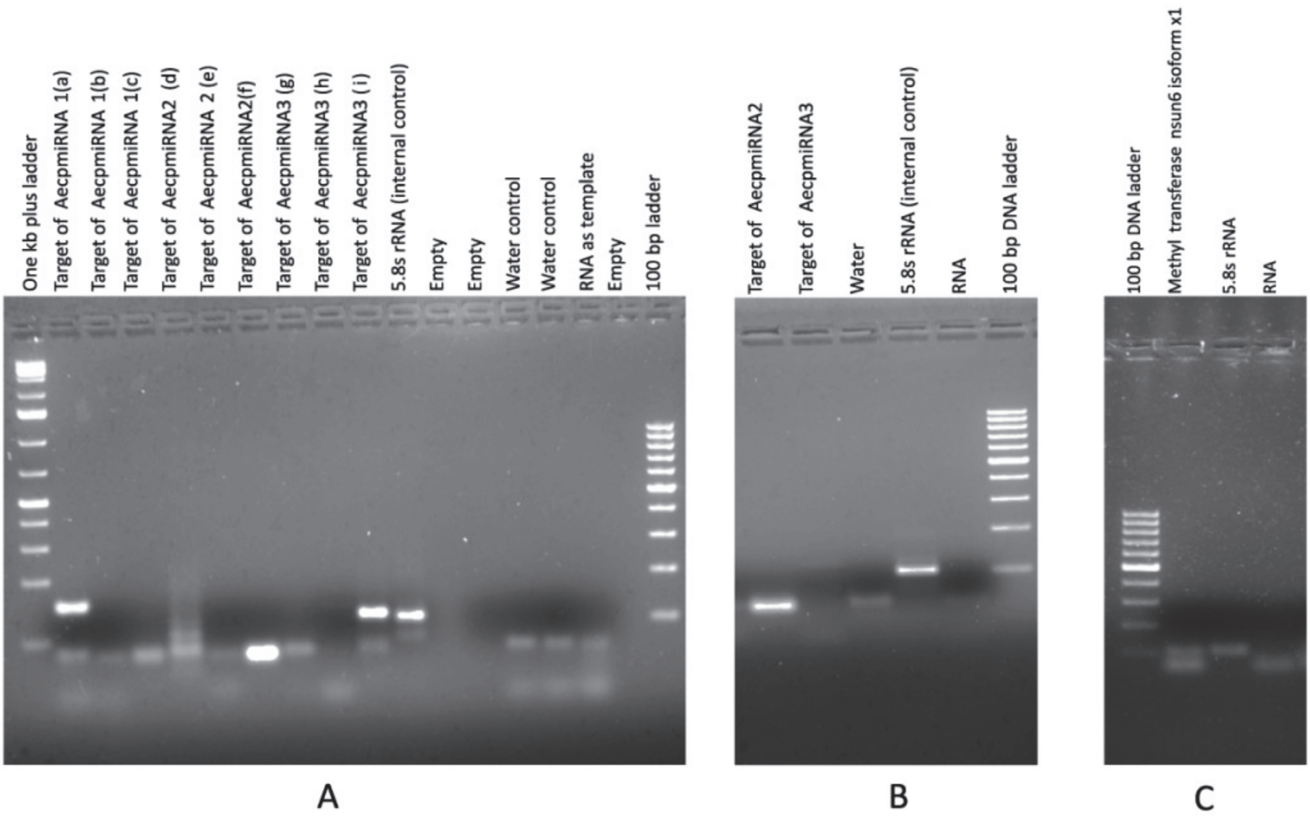

Fig. S5. RT-PCR for *A. esculentus* AecpmiRNA targets; (A) RT-PCR carried out at annealing temperature 60 °C: (a) Yth domain-containing family protein 2-like isoform × 1 (137 bp), (b) methyl transferase nsun6 isoform × 1 (100 bp), (c) E3 ubiquitin-protein ligase rglg × 1 (isoform × 1) (109 bp), (d) alfa-xylosidase 1-like (109 bp), (e) ethylene insensitive 3-like protein isoform (100 bp), (f) 187-kda microtubule associated protein (114 bp), (g) splicing factor 3b subunit 3-like (117 bp), (h) 3-Ketoacyl-coA thiolase5 (121 bp), (i) psbN (109 bp); to assure the amplification from *A. esculentus* cDNA, 5.8S rRNA as internal control was used and to neglect the false amplification negative controls (water and RNA) were used; (B) RT-PCR for AecpmiRNA2 and AecpmiRNA3 targets 187-kDa microtubule associated protein (114 bp) and 3-Ketoacyl-coA thiolase5 (121 bp), respectively carried out at annealing temperature 58 °C; (C) RT-PCR for Methyl transferase nsun6 isoform x1 (AecpmiRNA1 target) (100 bp) carried out at annealing temperature 55.9 °C
